# Towards EEG Biomarkers of Emotional Burnout Syndrome: gender related variations in functional connectivity under Resistance stage formation

**DOI:** 10.1192/j.eurpsy.2022.766

**Published:** 2022-09-01

**Authors:** S. Tukaiev, D. Harmatiuk, A. Popov, M. Makarchuk

**Affiliations:** 1 National Taras Shevchenko University of Kyiv, Institute Of Biology And Medicine, Kyiv, Ukraine; 2 Igor Sikorsky Kyiv Polytechnic Institute, Department Of Electronic Engineering, Kyiv, Ukraine

**Keywords:** functional connectivity, gender, emotional burnout, Resistance stage

## Abstract

**Introduction:**

The phenomenon of burnout generates the most interest due to relation to complete or partial disengagement of emotions, cognitive impairment, impairment of long-term and working memory. The neurophysiological mechanisms of emotional burnout remain insufficiently studied. Data related to gender specific characteristics of burnout formation are contradictory.

**Objectives:**

To establish the gender related EEG markers of burnout was our aim.

**Methods:**

621 volunteers (443 females) aged 18 to 24 years participated in this study. EEG was registered during the resting state (3 min, closed eyes condition). The interhemispheric and intrahemispheric average coherence across all EEG segments in all frequencies from 0.2-45 Hz was estimated. Psychological testing was performed before the registration of EEG. To determine the level of burnout formation the Boyko`s Syndrome of Emotional Burnout Inventory (SEB) was used.

**Results:**

The Resistance phase of emotional burnout was formed in 139 women and 42 men. Development of Resistance stage in female includes formation of new intrahemispheric connections predominantly in the left frontal region (alpha1,2,3-subbands) and the midline frontal-central axis (Fz-Cz, alpha1,2 and theta2-subbands). At the same time new intrahemispheric links in men under Resistance stage development are formed mainly in the right frontal region (alpha1,2,3-subbands).

**Conclusions:**

Connectivity patterns displayed gender-related variations that are associated with the difference in the alterations in the attention focusing, working memory, and emotional processes under burnout formation.

**Disclosure:**

No significant relationships.

